# Improving the Predictive Accuracy of the National Early Warning Score 2: Protocol for Algorithm Refinement

**DOI:** 10.2196/70303

**Published:** 2025-07-21

**Authors:** Chris Plummer, Cen Cong, Madison Milne-Ives, Lynsey Threlfall, Peta Le Roux, Edward Meinert

**Affiliations:** 1 Department of Cardiology Newcastle upon Tyne Hospitals NHS Foundation Trust Newcastle upon Tyne United Kingdom; 2 NIHR Newcastle Biomedical Research Centre Newcastle University Newcastle upon Tyne United Kingdom; 3 Translational and Clinical Research Institute Newcastle University Newcastle upon Tyne United Kingdom; 4 Centre for Health Technology School of Nursing and Midwifery University of Plymouth Plymouth United Kingdom; 5 Acute Medicine Newcastle upon Tyne Hospitals NHS Foundation Trust Newcastle upon Tyne United Kingdom; 6 Information Services Newcastle upon Tyne Hospitals NHS Foundation Trust Newcastle upon Tyne United Kingdom; 7 Department of Primary Care and Public Health Imperial College London London United Kingdom

**Keywords:** clinical deterioration, National Early Warning Score, performance, proof-of-concept

## Abstract

**Background:**

The National Early Warning Score 2 (NEWS2) has been widely adopted for predicting patient deterioration in health care settings using routinely collected physiological observations. The use of NEWS2 has been shown to reduce in-hospital mortality, but it has limited accuracy in the prediction of clinically important outcomes, especially over longer time periods.

**Objective:**

This project aims to improve the predictive accuracy of the NEWS2 scoring system, particularly its accuracy over more than 24 hours and its predictive value in older patients and children. It will investigate whether using the currently collected data differently and the inclusion of additional data would result in an improved algorithm.

**Methods:**

The study will use historical patient data from the Newcastle upon Tyne Hospitals NHS Foundation Trust, including observational data (eg, vital signs), BMI- related data, and other outcome-related variables (eg, mortality rates) to train and test an algorithm to predict the risk of key clinical outcomes, including mortality, intensive therapy unit admission, sepsis, and cardiac arrest, to demonstrate a proof of concept for a modified scoring system. The algorithm’s performance will be assessed based on its accuracy, precision, *F*_1_-score, area under the curve, and receiver operating characteristic curve.

**Results:**

The study is expected to start in April 2025. The findings are expected to be produced by the end of 2026 and will be disseminated at symposia, conferences, and in journal publications.

**Conclusions:**

The refined NEWS2 algorithm will address limited accuracy in predicting clinical deterioration beyond 24 hours in the original system by incorporating additional variables. Improved accuracy in the early detection of deterioration can lead to timely interventions, potentially reducing mortality and adverse clinical events. The enhanced algorithm also has the potential to be integrated into existing clinical decision support systems to facilitate health care professionals’ decision-making.

**International Registered Report Identifier (IRRID):**

PRR1-10.2196/70303

## Introduction

The National Early Warning Score 2 (NEWS2) system is a widely used tool for the systematic documentation and identification of clinical deterioration. It has been demonstrated to be effective at reducing in-hospital mortality [1-4], it facilitates effective communication between clinicians and enables timely interventions to improve patient outcomes, but NEWS2 has limited positive and negative predictive accuracy [5-7], particularly in predicting adverse events beyond 24 hours [8-10].

The use of digital technologies in health care presents an opportunity to evaluate whether the inclusion of additional routinely collected variables would improve the predictive accuracy of an early warning score and thus reduce patient risk. The same technologies could then be used to provide clinical decision support using artificial intelligence–derived algorithms that would not be practical using paper observation charts and health care records [1,11].

This further personalization of risk prediction is increasingly important in our aging population. Older adults are particularly vulnerable to sudden changes in physiological status [12], but this group was not included in the development of NEWS2 [13]. Improved predictive accuracy in this growing frail and vulnerable group could significantly improve patient outcomes [13-15].

In an evaluation of the performance of the original National Early Warning Score (NEWS), it demonstrated consistent predictive accuracy across multiple patient groups [5]. However, the performance of NEWS2 varies between care settings and between patients with different characteristics [11,16,17]. NEWS2 has been found to have significantly lower specificity, sensitivity, and positive predictive value for those who were at risk of type II respiratory failure, but higher specificity and positive predictive value for those with documented type II respiratory failure status, than the original NEWS [16]. The accuracy of NEWS has also been found to vary between care settings [17]. These observations suggest that the inclusion of individualized patient and care setting characteristics may improve the predictive accuracy, sensitivity, and specificity of the system and thus improve outcomes in a range of health care settings and in diverse patient groups.

To determine which variables may improve the risk prediction of NEWS2, we have reviewed 6 databases (CINAHL, PubMed, Embase, ScienceDirect, Cochrane Library, and Web of Science) [18]. Our preliminary findings show that demographic variables including age and ethnicity [6,19], trend data [20-22], and adjustments to component weighting [23,24] may improve the performance of NEWS2 in predicting clinical deterioration.

The principal aim of this work is to develop an algorithm that can improve the predictive accuracy and thus clinical value of the NEWS2 system. The objectives of this study are as follows:

1. To determine which additional variables have been previously used to enhance the accuracy of clinical early warning scores, including NEWS2.
2. To collect a large set of patient data that links observations, demographic, and admissions data with key deterioration-related outcomes.
3. To use these data to train an artificial intelligence model to determine which variables, and what weightings, provide optimal predictive accuracy.
4. To test the accuracy, sensitivity, and specificity of the algorithm.
5. To consider how the new model could be applied across a wide range of health care settings, including different physiological observation equipment, different electronic patient record systems, or paper-based clinical record systems.

## Methods

### Overview

The study is a retrospective cohort study using historical, anonymized patient data to explore ways in which a new alerting system could improve NEWS2. The Newcastle Hospitals National Health Service (NHS) Foundation Trust has an established information governance system for the use of anonymized patient data for research. This study will use historical, anonymized hospital inpatient and emergency care data from the Newcastle upon Tyne Hospitals NHS Foundation Trust, collected routinely over 6 years (December 2018 to June 2024).

Patient and public involvement (PPI) refers to research being carried out “with” or “by” members of the public rather than “to,” “about” or “for” them [25]. To support PPI in research, we will recruit PPI members through the Newcastle BRC Informatics and Precision Care for an Ageing Population theme and Newcastle University’s Clinical Trials Unit. They will be included in all project management group meetings and will provide their lived experience with hospital monitoring to highlight any concerns or barriers they foresee in the adoption and implementation of a modified NEWS system. At least 1 older adult will be included to ensure that we are including a perspective that has been missing in the development of previous NEWS systems. A logic diagram of the study design is presented in Multimedia Appendix 1.

The model will be developed following the recommended process of building a predictive model with machine learning [26,27]. The research question and a clear goal of building a model will be first defined. The following types of data (Table 1) will then be obtained from the Trust.

**Table 1:** Input variables.

Type	Input variables
General	Patient ID
Demographic data	Age Biological sex Ethnicity
Observational data (vital signs and other measures)	Observation date, time, and location Encounter: ID and type Heart rate Temperature Respiratory rate Need of oxygen therapy Level of consciousness or new confusion (Alert, Verbal, Pain, or Unresponsive score) Blood pressure (systolic and diastolic) Paired value One (lying blood pressure) Paired value Two (standing blood pressure) Oxygen therapy percentage—inspired oxygen fraction (FiO2) Oxygen saturation (SpO2) Nursing concern Urine output Pain score Mask code (oxygen therapy device used) Partial Obs set (NEWS2^a^ variables) Partial Obs set (additional variables)
BMI data (in separate data file)	Height (numeric, units, date, and time) Weight (numeric, units, date, and time) BMI (score date and time)
Outcome-related data	Mortality Cardiac arrest Resuscitation Unplanned admission to critical care: include either intensive therapy unit or high-dependency unit Sepsis NEWS2 score Northumbria Healthcare NHS^b^ Foundation Trust risk rating (NuTH)- overwrite thresholds (NuTH specific)

^a^NEWS2: National Early Warning Score.

^b^NHS: National Health Service.

The data will then be deidentified before the research team can access it within a secure project environment in the Trust’s IT network for training and testing the algorithm. Data will also be prepared by handling missing values and any inconsistencies or imbalances. Deidentified data from the Trust’s clinical data marts, including patient observations, admissions information, demographic data, and outcomes data, will be divided into training and testing subsets. A decision will then be made on whether to test the performance of multiple existing models with additional data or improve the performance of 1 model (eg, eXtreme Gradient Boosting). Using these datasets, we will train and test an algorithm that optimizes the variables and their weightings to predict the risk of key clinical outcomes, including mortality, intensive therapy unit admission, sepsis, and cardiac arrest, to demonstrate a proof of concept for a modified scoring system.

The following metrics will be used to evaluate the model’s performance. Definitions and equations are obtained from the literature [28,29].

1. Accuracy: the number of correct predictions divided by the total number of predictions.
2. Precision: the ratio of true positives and total positives predicted.
3. F1-score: the harmonic mean of precision and recall.
4. Area under the curve (AUC): the probability that the model, if given a randomly chosen positive and negative example, will rank the positive higher than the negative. The closer the AUC is to 1.0, the better the model’s ability to separate classes from each other.
5. Receiver-operating characteristic curve: this curve is typically plotted in terms of true positive rate and false positive rate. It is drawn by calculating the true positive rate and the false positive rate at every possible threshold (in practice, at selected intervals), then graphing the true positive rate over the false positive rate.

The performance of the model will be optimized through hyperparameter tuning and configuration refining. The final model will be tested with the test set against predetermined metrics. Depending on funding availability, the model may be validated using external collaborator’s datasets.

### Ethical Considerations

This study (Integrated Research Application System Project ID 344472) has received ethical approval from the UK Health Research Authority Research Ethical Committee (Rec reference 25/WA/0060). Informed consent and compensation do not apply to the study, as no human participants will be involved. A data scientist at the Trust will exclude all data from patients who have opted out of their data being used for research purposes through local or national processes. All patient data used in this project are fully deidentified by the NHS before being accessed by the research team, ensuring no personal information about participants is collected. Data sharing is restricted to authorized team members with NHS-approved credentials and access is managed through secure NHS systems with strict monitoring. During the project, data analysis will be conducted within password-protected environments. After the project, data will be securely archived or deleted following NHS policies. Patients will not be identified in any way in the findings to ensure participant confidentiality.

## Results

Algorithm development has not yet commenced as of February 26, 2025. Work is expected to start in April 2025 and findings are expected to be produced by December 2026. By the end of this study, we will have trained and tested an algorithm based on historical patient data from the Newcastle upon Tyne Hospitals NHS Foundation Trust. This will provide a proof-of-concept of whether and which additional data can improve the predictive accuracy of the NEWS2 system.

After refinement, the algorithm is expected to be able to predict clinical disease deterioration accurately. This will be assessed by comparing the algorithm’s predictions against observed outcomes, including mortality, cardiac arrest, unplanned admissions to critical care units (intensive therapy units or high-dependency units), resuscitation events, and the occurrence or progression of sepsis. A summary of outcome items and measures is listed in Table 2.

**Table 2:** Outcome items and measures.

Item	Definition	Measure	Expected result
Mortality	Whether the patient survives within a specified time frame	Quantitative: mortality rate	High accuracy and precision against patient records
Cardiac arrest	Sudden loss of all heart activity	Quantitative: number of correctly predicted cardiac arrest cases	High accuracy and precision against documented cardiac arrest cases
Unplanned critical care admissions	Whether the patient requires admission to intensive care	Quantitative: number of correctly predicted admissions	High accuracy and precision against observed admissions to critical care units
Resuscitation events	Process of reviving acutely ill patient	Quantitative: number of correctly predicted resuscitation events	High accuracy and precision against the actual number of events
Sepsis	Life-threatening reaction to an infection	Quantitative: number of correctly predicted sepsis events	High accuracy and precision against documented sepsis cases

## Discussion

### Principal Findings

The anticipated results of the study include the algorithm’s increased predictive accuracy. There are 2 main ways in which this could improve patient outcomes (1) improving the system’s ability to accurately predict patients who will deteriorate within and beyond 24 hours would allow health care professionals to optimize their care and prevent deterioration and (2) reducing false positive alerts would allow care to be concentrated where it will achieve the greatest benefit.

### Comparison to Prior Work

Several studies have developed machine learning algorithms (eg, logistic regression, support vector machine, gradient boosting decision tree, and XGBoost) to predict clinical deterioration in emergency departments, such as in-hospital cardiac arrest, clinical deterioration, and mortality, using historical prehospital data or emergency department data in China and Korea [30-32]. While these algorithms achieved high accuracy (AUC over 0.8) in predicting clinical deterioration in emergency department patients, their relevance and potential generalizability to different population demographics and health care contexts remain unknown. This study uses historical patient data from a UK-based NHS Trust, making the algorithm more relevant to the UK health care system. In addition, it includes more demographic variables (ie, age and ethnicity) and BMI-related data to inform a more comprehensive risk assessment. However, this study still requires validation with external datasets to increase its generalizability.

### Future Directions

Patient and Public Involvement and Engagement members will help guide the dissemination of the results of the study to the population through publicly accessible forums. Following this work, we will test the new system on data in other NHS Trusts across the United Kingdom to assess its generalizability. In 2026, we will conduct implementation studies in Newcastle and then in other NHS Trusts to assess the adoption of the system and gather initial data on patient outcomes to construct a learning health care system to evaluate the real-world performance of the algorithm and facilitate continual improvement. By 2029, we aim to implement the system in NHS Trusts across the United Kingdom and conduct large-scale trials to evaluate its effectiveness in improving patient outcomes.

### Strengths and Limitations

This work highlights the importance of investigating the use of additional clinical variables to those used in NEWS2 in the development of a National Early Warning Score. The study design was informed by an evidence synthesis of the literature. Despite these strengths, this project also has limitations. Some retrospective datasets may be of low quality or incomplete. Furthermore, external validation will be needed to test algorithm generalizability.

### Conclusion

This work has identified potential areas of improvement to the NEWS2 system and outlined a project for improving its ability to predict adverse outcomes through the use of additional patient data or changes to the scoring process. The project’s outputs will be shared widely to facilitate collaboration between health care professionals, researchers, and policymakers to support the implementation of improved clinical early warning systems in diverse populations.

## Appendix

Multimedia Appendix 1 Study logic diagram.

Multimedia Appendix 2 Peer review report from the NIHR Newcastle Biomedical Research Centre (National Institute for Health and Care Research, UK).

## Abbreviations

AUC: area under the curve
NEWS: National Early Warning Score
NEWS2: National Early Warning Score 2
NHS: National Health Service
PPI: patient and public involvement
XGBoost: extreme gradient boosting

## Data Availability

Data sharing is not applicable to this article as no data sets were generated or analyzed during this study.
