# Supplementary figures and images for: Improving the Predictive Accuracy of the National Early Warning Score 2: Protocol for Algorithm Refinement
Source: JMIR Res Protoc. 2025 Jul 21;14:e70303. doi: 10.2196/70303 (PMC12322607; doi:10.2196/70303)

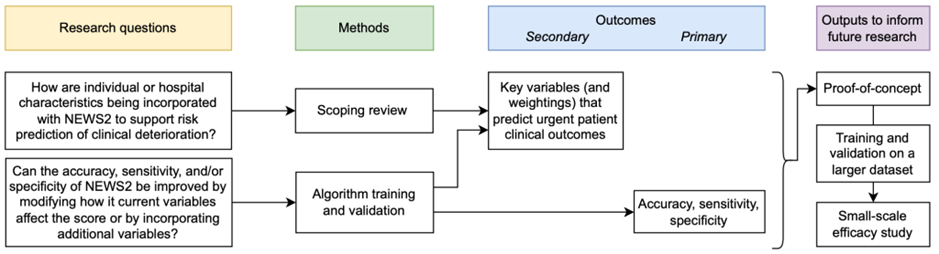

Supplement: Multimedia Appendix 1 [file resprot_v14i1e70303_app1.png]
