# Supplementary material for: Improving the Predictive Accuracy of the National Early Warning Score 2: Protocol for Algorithm Refinement
Source: JMIR Res Protoc. 2025 Jul 21;14:e70303. doi: 10.2196/70303 (PMC12322607; doi:10.2196/70303)
Supplement: Multimedia Appendix 2 [file resprot_v14i1e70303_app2.pdf]

NIHR | Newcastle Biomedical Research Centre

NIHR Newcastle BRC Interdisciplinary Research Call

Overarching Criteria

Application supports BRC remit of high quality early translational research and experimental medicine that benefits patients

Additional Stage 1 scoring criteria

- Do the co-applicants represent more than one discipline?
- Does the application fall within the remit of at least one BRC theme?
- Does the application align with one of the BRC's IDR priority areas?
- Does the applicant propose cross-theme collaborations or opportunities for interdisciplinarity?
- Does the applicant describe a strong research proposal?
- Does the applicant intend to collaborate with industry partners?
- Does the proposal have strong PPIE plans?
- Does the applicant describe realistic and achievable outputs?

Summary of scoring and reviews

Lead applicant - Dr Chris Plummer

Short title - Improving NEWS2 predictive accuracy

Application value - £49,757

NIHR | Newcastle Biomedical Research Centre

Stage/Review	Comments
**Stage 1**	Ranked 4th out of 20 applications.<br>Digital approach to outcome prediction based on accessing NuTH data. Strong IDR team with clear clinical leads, access to data and aglorithm development. Focus on improving the NEWS2 algorithm particulalry for older patients whose demographic was under-represented in the original NEWS development.
**Reviewer 1**	Interesting and important subject with an excellent team. The problem is the lack of detail. The whole thing is extremely vague, and it is very hard to work out exactly what they will do. The ageing link feels tokenistic. How they will incorporate time as a variable, how they will assess accuracy of sepsis diagnoses (for example), the sample size, the level of acceptable predictive accuracy etc, are unclear. Their approach can only improve on NEWS2, but they never state how much better a new score ought to be.
**Reviewer 2**	Strong team. Don’t fully understand the detail of how the project will involve but sounds very interesting and clear potential for national roll out/impact.
**Reviewer 3 (PPIE)**	No issues
